# 10-(Prop-1-yn-1-yl)-10*H*-phenothia­zine

**DOI:** 10.1107/S1600536812036537

**Published:** 2012-08-25

**Authors:** Satoru Umezono, Tsunehisa Okuno

**Affiliations:** a Department of Material Science and Chemistry, Wakayama University, Sakaedani, Wakayama 640-8510, Japan

## Abstract

In the title compound, C_15_H_11_NS, the asymmetric unit comprises one half-mol­ecule; a mirror plane passes through the S atom, the ynamine fragment, the methyl C atom and one methyl H atom. The phenothia­zine moiety has a butterfly conformation and the central six-membered ring has a boat conformation. The dihedral angle between the benzene rings is 149.40 (4)°. The crystal structure is stabilized by van der Waals inter­actions.

## Related literature

For related structures of phenothia­zine compounds, see: Okuno *et al.* (2006 ▶); Tabata & Okuno (2012 ▶). For the preparation of the title compound, see: Zaugg *et al.* (1958 ▶).
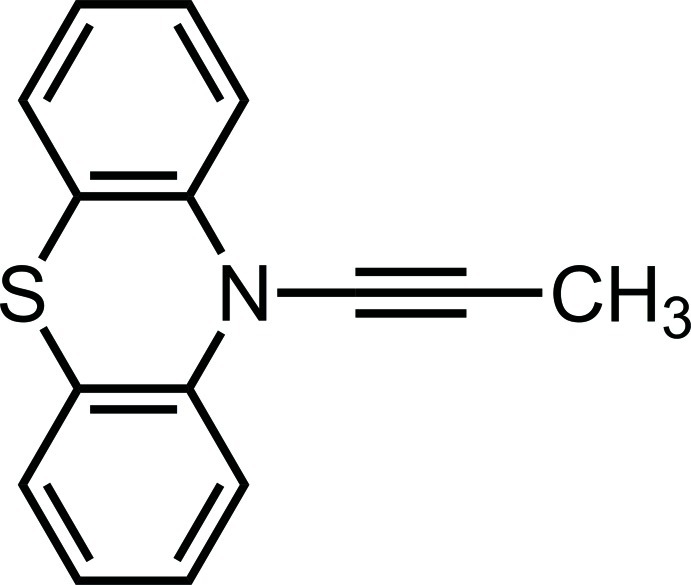


## Experimental

### 

#### Crystal data

C_15_H_11_NS
*M* *_r_* = 237.31
Orthorhombic, 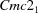

*a* = 14.717 (6) Å
*b* = 10.631 (4) Å
*c* = 7.375 (3) Å
*V* = 1153.9 (8) Å^3^
*Z* = 4
Mo *K*α radiation
μ = 0.25 mm^−1^
*T* = 93 K
0.12 × 0.10 × 0.08 mm

#### Data collection

Rigaku Saturn724+ diffractometer
Absorption correction: numerical (*NUMABS*; Rigaku, 1999 ▶) *T*_min_ = 0.959, *T*_max_ = 0.980
4889 measured reflections
1486 independent reflections
1441 reflections with *I* > 2σ(*I*)
*R*_int_ = 0.019

#### Refinement

*R*[*F* ^2^ > 2σ(*F* ^2^)] = 0.025
*wR*(*F* ^2^) = 0.065
*S* = 1.08
1486 reflections
87 parameters
1 restraint
H-atom parameters constrained
Δρ_max_ = 0.20 e Å^−3^
Δρ_min_ = −0.16 e Å^−3^
Absolute structure: Flack (1983 ▶), 670 Friedel pairs
Flack parameter: −0.01 (6)

### 

Data collection: *CrystalClear* (Rigaku, 2008 ▶); cell refinement: *CrystalClear*; data reduction: *CrystalClear*; program(s) used to solve structure: *SHELXS97* (Sheldrick, 2008 ▶); program(s) used to refine structure: *SHELXL97* (Sheldrick, 2008 ▶); molecular graphics: *ORTEP-3* (Farrugia, 1997 ▶); software used to prepare material for publication: *SHELXL97*.

## Supplementary Material

Crystal structure: contains datablock(s) global, I. DOI: 10.1107/S1600536812036537/bx2425sup1.cif

Structure factors: contains datablock(s) I. DOI: 10.1107/S1600536812036537/bx2425Isup2.hkl

Supplementary material file. DOI: 10.1107/S1600536812036537/bx2425Isup3.cml

Additional supplementary materials:  crystallographic information; 3D view; checkCIF report

## Figures and Tables

**Fig. 1.**
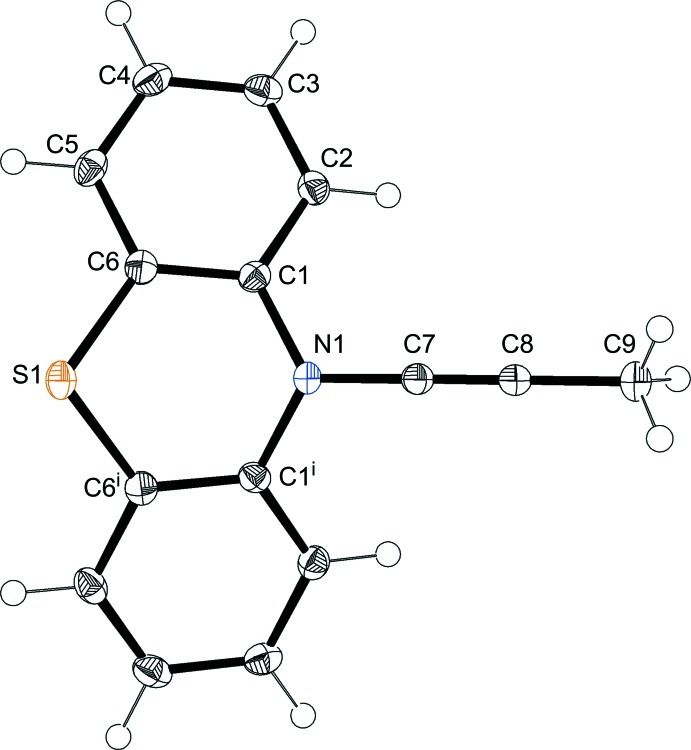
ORTEP view of the title compound with atom-numbering scheme. Displacement ellipsoids are drawn at the 50% probability level and H atoms are shown as small spheres. [Symmetry code: (i) -x, y, z.]

## supplementary crystallographic information

### Comment

Ynamines, where amino groups connect to acetylene groups, are known to be unstable because of their high reactivity. The title compound, C_15_H_11_NS, is the first ynamine compound which was prepared accidentally (Zaugg *et al.*, 1958). The asymmetric unit comprises one half-molecule; a mirror plane passes through the S, ynamine fragment, one C atom of methyl group and one H atom of methyl group. The bond distances and angles are comparable with others reported ynamines (Okuno *et al.*, 2006; Tabata *et al.*, 2012). The crystal structure is stabilized by van der Waals interactions. The phenothiazine moiety has a butterfly conformation, and the central six-membered ring has a boat conformation. The dihedral angle between two benzene rings is 149.40 (4)°.

### Experimental

The title compound was prepared according to a published procedure (Zaugg *et al.*, 1958). The single crystals with sufficient quality for X-ray analysis were obtained by slow concentration of a methanol solution.

### Refinement

The C-bound H atoms were placed at ideal positions and were refined as riding on their parent C atoms. *U*_iso_(H) values of H2—H5 atoms were set at 1.2*U*_eq_(parent atom).

### Crystal data

**Table:**

C_15_H_11_NS	*F*(000) = 496
*M**_r_* = 237.31	*D*_x_ = 1.366 Mg m^−^^3^
Orthorhombic, *C**m**c*2_1_	Mo *K*α radiation, λ = 0.71075 Å
Hall symbol: C 2c -2	Cell parameters from 2342 reflections
*a* = 14.717 (6) Å	θ = 2.4–31.2°
*b* = 10.631 (4) Å	µ = 0.25 mm^−^^1^
*c* = 7.375 (3) Å	*T* = 93 K
*V* = 1153.9 (8) Å^3^	Block, colorless
*Z* = 4	0.12 × 0.10 × 0.08 mm

### Data collection

**Table:**

Rigaku Saturn724+ diffractometer	1441 reflections with *I* > 2σ(*I*)
Detector resolution: 28.445 pixels mm^-1^	*R*_int_ = 0.019
ω scans	θ_max_ = 28.5°, θ_min_ = 2.4°
Absorption correction: numerical (*NUMABS*; Rigaku, 1999)	*h* = −19→16
*T*_min_ = 0.959, *T*_max_ = 0.980	*k* = −14→12
4889 measured reflections	*l* = −9→9
1486 independent reflections	

### Refinement

**Table:**

Refinement on *F*^2^	Secondary atom site location: difference Fourier map
Least-squares matrix: full	Hydrogen site location: inferred from neighbouring sites
*R*[*F*^2^ > 2σ(*F*^2^)] = 0.025	H-atom parameters constrained
*wR*(*F*^2^) = 0.065	*w* = 1/[σ^2^(*F*_o_^2^) + (0.0354*P*)^2^ + 0.514*P*] where *P* = (*F*_o_^2^ + 2*F*_c_^2^)/3
*S* = 1.08	(Δ/σ)_max_ < 0.001
1486 reflections	Δρ_max_ = 0.20 e Å^−^^3^
87 parameters	Δρ_min_ = −0.16 e Å^−^^3^
1 restraint	Absolute structure: Flack (1983), 670 Friedel pairs
Primary atom site location: structure-invariant direct methods	Flack parameter: −0.01 (6)

### Special details

**Table:**

Refinement. Refinement was performed using all reflections. The weighted *R*-factor (*wR*) and goodness of fit (*S*) are based on *F*^2^. *R*-factor (gt) are based on *F*. The threshold expression of *F*^2^ > 2.0 σ(*F*^2^) is used only for calculating *R*-factor (gt).

### Fractional atomic coordinates and isotropic or equivalent isotropic displacement parameters (Å^2^)

**Table:**

	*x*	*y*	*z*	*U*_iso_*/*U*_eq_	
S1	0.0000	0.03514 (4)	−0.00174 (4)	0.01839 (11)	
N1	0.0000	0.28986 (13)	0.1771 (2)	0.0165 (3)	
C1	0.08381 (8)	0.22366 (10)	0.19225 (16)	0.0153 (2)	
C2	0.15834 (8)	0.27842 (11)	0.27878 (17)	0.0176 (2)	
H2	0.1524	0.3585	0.3346	0.021*	
C3	0.24154 (8)	0.21576 (12)	0.28336 (18)	0.0201 (3)	
H3	0.2926	0.2542	0.3393	0.024*	
C4	0.24978 (8)	0.09669 (12)	0.20594 (18)	0.0212 (3)	
H4	0.3067	0.0545	0.2075	0.025*	
C5	0.17460 (9)	0.03964 (11)	0.12637 (17)	0.0189 (3)	
H5	0.1797	−0.0430	0.0783	0.023*	
C6	0.09172 (8)	0.10329 (11)	0.11690 (15)	0.0163 (2)	
C7	0.0000	0.41727 (16)	0.1900 (2)	0.0164 (3)	
C8	0.0000	0.52987 (15)	0.1916 (3)	0.0171 (3)	
C9	0.0000	0.66718 (16)	0.1911 (3)	0.0218 (4)	
H9A	0.0000	0.7026	0.3042	0.045 (8)*	
H9B	−0.0435	0.7017	0.1183	0.050 (6)*	

### Atomic displacement parameters (Å^2^)

**Table:**

	*U*^11^	*U*^22^	*U*^33^	*U*^12^	*U*^13^	*U*^23^
S1	0.0226 (2)	0.01654 (18)	0.01607 (19)	0.000	0.000	−0.00403 (15)
N1	0.0160 (7)	0.0124 (6)	0.0212 (7)	0.000	0.000	−0.0023 (5)
C1	0.0150 (5)	0.0154 (5)	0.0155 (5)	0.0004 (4)	0.0028 (4)	0.0021 (4)
C2	0.0188 (6)	0.0159 (5)	0.0180 (5)	−0.0016 (5)	0.0009 (5)	0.0001 (4)
C3	0.0165 (6)	0.0225 (6)	0.0212 (6)	−0.0021 (5)	0.0001 (5)	0.0039 (4)
C4	0.0178 (5)	0.0209 (6)	0.0249 (6)	0.0043 (5)	0.0040 (5)	0.0060 (5)
C5	0.0218 (6)	0.0159 (5)	0.0189 (6)	0.0030 (4)	0.0061 (5)	0.0021 (4)
C6	0.0183 (5)	0.0160 (5)	0.0144 (5)	−0.0007 (4)	0.0027 (4)	0.0011 (4)
C7	0.0146 (7)	0.0172 (8)	0.0175 (7)	0.000	0.000	−0.0018 (6)
C8	0.0143 (7)	0.0165 (8)	0.0206 (8)	0.000	0.000	−0.0014 (6)
C9	0.0232 (9)	0.0142 (7)	0.0278 (9)	0.000	0.000	−0.0011 (7)

### Geometric parameters (Å, º)

**Table:**

S1—C6^i^	1.7642 (13)	C3—H3	0.9500
S1—C6	1.7643 (13)	C4—C5	1.3914 (19)
N1—C7	1.358 (2)	C4—H4	0.9500
N1—C1	1.4246 (14)	C5—C6	1.3966 (17)
N1—C1^i^	1.4246 (14)	C5—H5	0.9500
C1—C2	1.3961 (17)	C7—C8	1.197 (3)
C1—C6	1.3999 (17)	C8—C9	1.460 (2)
C2—C3	1.3944 (17)	C9—H9A	0.9152
C2—H2	0.9500	C9—H9B	0.9126
C3—C4	1.3940 (18)		
			
C6^i^—S1—C6	99.84 (8)	C5—C4—H4	120.0
C7—N1—C1	119.16 (7)	C3—C4—H4	120.0
C7—N1—C1^i^	119.16 (7)	C4—C5—C6	120.29 (11)
C1—N1—C1^i^	119.97 (14)	C4—C5—H5	119.9
C2—C1—C6	119.82 (11)	C6—C5—H5	119.9
C2—C1—N1	120.67 (11)	C5—C6—C1	119.71 (11)
C6—C1—N1	119.50 (11)	C5—C6—S1	119.61 (9)
C3—C2—C1	120.12 (11)	C1—C6—S1	120.57 (9)
C3—C2—H2	119.9	C8—C7—N1	176.53 (19)
C1—C2—H2	119.9	C7—C8—C9	179.3 (2)
C4—C3—C2	120.02 (11)	C8—C9—H9A	114.2
C4—C3—H3	120.0	C8—C9—H9B	113.8
C2—C3—H3	120.0	H9A—C9—H9B	111.8
C5—C4—C3	119.96 (11)		
			
C7—N1—C1—C2	21.9 (2)	C4—C5—C6—S1	174.51 (9)
C1^i^—N1—C1—C2	−143.06 (11)	C2—C1—C6—C5	−1.00 (17)
C7—N1—C1—C6	−157.02 (14)	N1—C1—C6—C5	177.97 (12)
C1^i^—N1—C1—C6	38.0 (2)	C2—C1—C6—S1	−177.19 (9)
C6—C1—C2—C3	2.76 (17)	N1—C1—C6—S1	1.78 (15)
N1—C1—C2—C3	−176.20 (12)	C6^i^—S1—C6—C5	152.23 (7)
C1—C2—C3—C4	−1.81 (18)	C6^i^—S1—C6—C1	−31.58 (13)
C2—C3—C4—C5	−0.91 (18)	C1—N1—C7—C8	97.44 (13)
C3—C4—C5—C6	2.68 (18)	C1^i^—N1—C7—C8	−97.44 (13)
C4—C5—C6—C1	−1.72 (18)	N1—C7—C8—C9	0.00 (5)

Symmetry code: (i) −*x*, *y*, *z*.
